# Painless Left Scrotal Mass: A Rare Case of Paratesticular Angiofibroma

**DOI:** 10.7759/cureus.24286

**Published:** 2022-04-19

**Authors:** Mustafa T Owaidah, Mohamad Bakir, Maher Moazin, Naif Aldaham, Saad R Alqasem, Abdullah Alfakri, Sami Almustanyir

**Affiliations:** 1 College of Medicine, Alfaisal University, Riyadh, SAU; 2 Urology, King Fahad Medical City, Riyadh, SAU; 3 Urology, Prince Sattam Bin Abdulaziz University, Alkharj, SAU; 4 Internal Medicine, Ministry of Health, Riyadh, SAU

**Keywords:** case report, mesenchymal tumor, paratesticular tumors, cellular angiofibroma, amf-like tumors, angiomyofibroblastoma-like tumors, angiofibromas

## Abstract

Angiofibromas, also known as angiomyofibroblastoma-like tumors or AMF-like tumors, refer to a collection of rare, benign yet highly cellular tumors of the vulva, scrotum, perineum, or inguinal region. In this paper, we present a 34-year-old Saudi man who presented with findings of a testicular tumor on physical examination and imaging and tested negative for all the markers associated with testicular tumors.

## Introduction

Paratesticular tumors are rare intrascrotal tumors, that arise from the epididymis, spermatic cord, and its coverings [1]. The first record of such a pathology was made in 1997 by Nucci et al. in the vulvar regions of four middle-aged women [2], while Laskin et al. first documented this pathology in 11 males in 1998, which were then labeled "Angiomyofibroblastoma-like tumors" [3]. Epidemiological data is scarce, with most of the evidence pointing toward ages 40 and above in both sexes. The pathogenesis of these tumors is still undetermined. We present the case of a 34-year-old man who presented with a testicular mass with no significant medical history.

## Case presentation

A 34-year-old Saudi male patient was referred to us from another hospital due to a two-year history of a painless left testicular mass. He is married with four healthy children. He had no significant family history, trauma, undescended testis, infections, or other risk factors that could have contributed to his condition.

**Figure 1:**
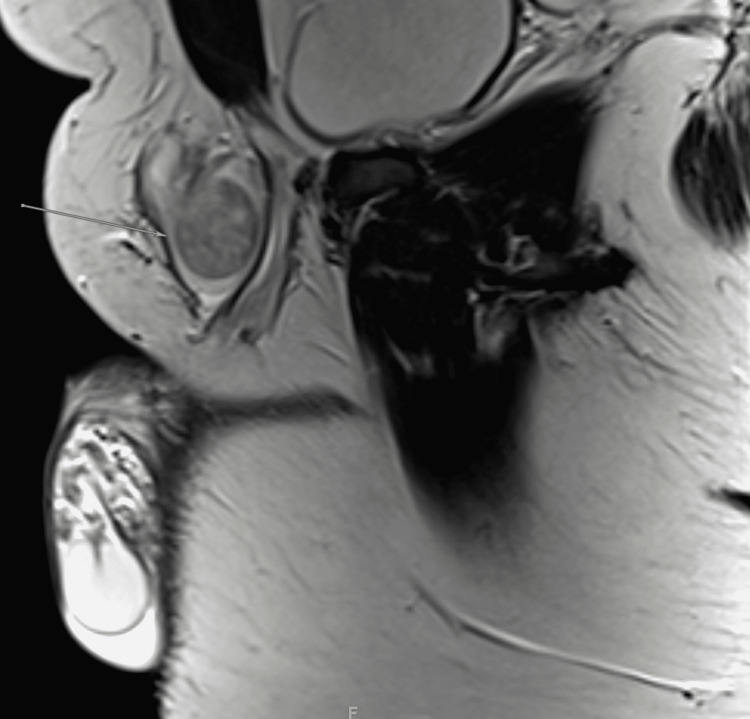
Sagittal T1-weighted MRI. Well-defined left inguinal canal lesion abutting the anterior aspect of the spermatic cord. The mass measured 3 x 3 x 5.5 cm in maximum anteroposterior, transverse, and craniocaudal diameter, respectively. The lesion demonstrates low intensity

**Figure 2:**
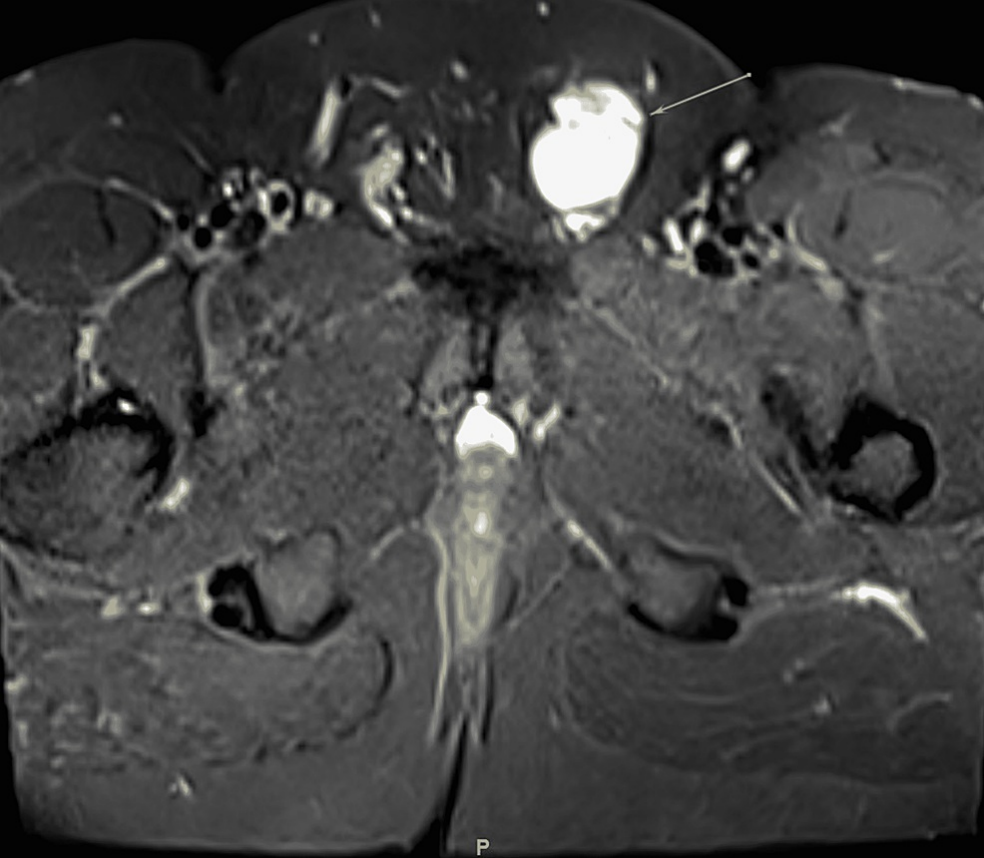
Cross-section T2-weighted MRI. No internal fatty or fibrous component. There is an intermediate to high signal intensity with intense homogeneous enhancement postcontrast administration. Unremarkable appearance of bilateral testicles and epididymis. No enlarged inguinal lymph nodes. Unremarkable urinary bladder, seminal vesicles, and prostate gland.

On physical examination, his vital signs were within the normal range. General, respiratory, cardiac, abdominal, and musculoskeletal examinations were normal. However, on genital examination, a large left testicular mass was noted. It involved the spermatic cord and had an oval shape on palpation. The right testicle, however, was of normal size, and lymphadenopathy was not observed. Ultrasonography revealed a testicular mass. Magnetic resonance imaging (MRI) was done with both T1 (Figure 1) and T2-weighted imaging (Figure 2), which showed an indeterminate hypervascular left inguinal canal mass abutting the anterior aspect of the spermatic cord. Tumor markers including beta-human chorionic gonadotropin, alpha-fetoprotein, and lactate dehydrogenase were tested, and all yielded negative results. A provisional diagnosis of paratesticular tumor was made, and the patient was sent for surgery.

**Figure 3:**
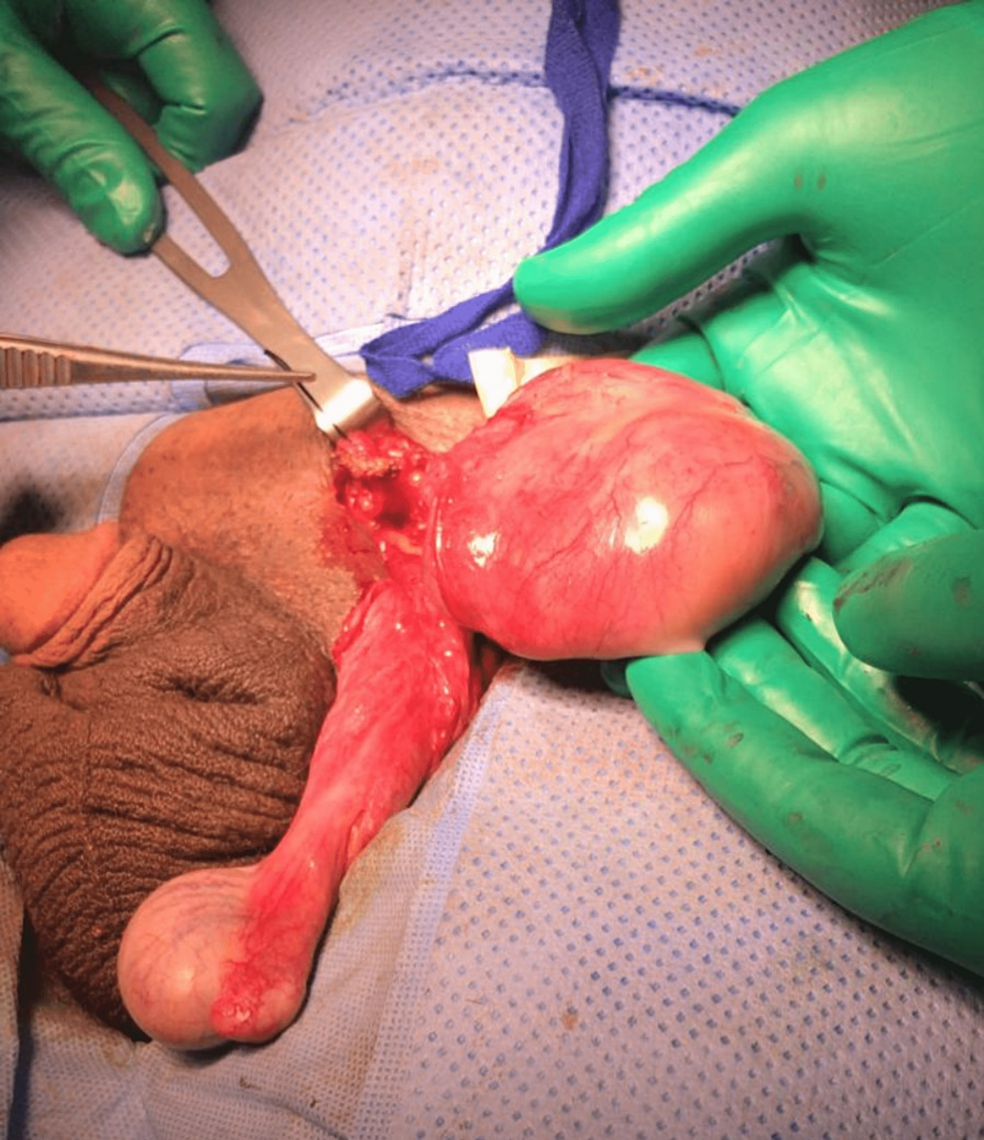
Gross image showing the paratesticular mass. The mass is located on the spermatic cord.

In the operating room, the patient was placed in the supine position and was administered general anesthesia. A left inguinal skin incision was made between the ipsilateral pubic tubercle and anterosuperior iliac spine, and the mass was found to be paratesticular with a hydrocele, contrary to the ultrasound image. The mass was isolated from the core and sent to the pathology department for further evaluation, and was determined to be a benign angiofibroma. The hydrocele sac was carefully freed from the surrounding tissue with a moist sponge to cleanly expose the partial layer of the tunica vaginalis (Figure 3). The hydrocele was incised and the fluid was aspirated. The testis, epididymis, and spermatic cord were inspected and found to be intact without any evidence of injury. They were carefully placed back into the scrotum in their normal anatomical position ensuring that the spermatic cord was not twisted. The findings revealed a left paratesticular mass with a left hydrocele. There were no complications, and the patient had an uneventful postoperative course.

On postoperative day two, the patient appeared to be recovering uneventfully, with no active issues or left testicular pain. His last recorded vital signs were within the normal range (Table1). He was scheduled for a follow-up after two weeks for wound assessment. On follow-up, he was mobilizing well and exhibited complete wound healing with no evidence of pain or discharge.

**Table 1:** Patient's vital signs on the second postoperative day.

Vital sign	Patient’s result	Reference range
Temperature	36.6°C (Axillary)	36.6°C to 37°C
Heart rate	82 beat per minute	60-100 beats per minute
Respiratory rate	20 breaths per minute	16-20 breaths per minute
Blood pressure	114/68 mm Hg	120/80 mm Hg
Oxygen saturation	100 % on room air.	95-100% on room air

## Discussion

AMF-like tumors are rare, benign neoplasms of mesenchymal origin. The most affected regions are the inguinal scrotal and vulvovaginal regions [4]. As previously stated, the tumor was first described in 1997 as a cellular angiofibroma. The name was changed to AMF-like tumor in 1998. Epidemiological data is limited because it is a rare condition. However, according to most of the literature, affected females are in their fifth decade and affected males are in their seventh decade [5]. This neoplasm has a wide range of differential diagnoses, including epithelioid leiomyoma, cellular angiofibroma, aggressive angiomyxoma, and angiomyofibroblastoma.

Most patients present asymptomatically with a painless mass, although some may experience mild-to-moderate pain. Grossly, the masses appear as well-circumscribed nodules with a soft to rubbery consistency and a gray-pink-brown cut surface. They have an average size of 5 cm in women and 12 cm in men. Histologically, the tumors appear to be well-circumscribed with a high degree of cellular variety, observable as areas of hyper- and hypocellularity, the latter of which is associated with stromal edema [6]. The cells vary in shape, ranging from rounded to spindle-shaped, with eosinophilic cytoplasm. Vessel wall hyalinization is also associated with this neoplasm but is mostly seen in postmenopausal patients with decreased edema and increased fibrosis.

The imaging modality used for this neoplasm was testicular ultrasonography, which is the best initial test. It revealed hypoechoic lesions. MRI can also be used for further evaluation. However, radiological findings are not very specific and do not significantly contribute to the differential diagnosis. In terms of immunohistochemistry, the tumor can express vimentin or desmin, both of which have strong expression, except in postmenopausal patients. Estrogen receptor, progesterone receptor, smooth muscle actin, and CD34 can also be expressed in this neoplasm [6].

The pathogenesis is still unknown; however, multiple hypotheses have been proposed, including the involvement of a monoallelic deletion of *RB1* and *FOX1* [7]. Both genes reside within chromosome 13q14, which is highly linked to the development of the disease. The treatment of choice is local excision with negative margins. The probability of recurrence is very low.

For male patients who present with a paratesticular mass, an extensive clinical evaluation is required, and physicians should be aware of this entity in order to avoid misdiagnosis and over-treatment of patients, as local excision can preserve the testicle and has favorable outcomes.

## Conclusions

AMF-like tumors are rare benign paratesticular tumors. They share the same clinical picture as some malignant tumors of the testicles, or even certain types of hernias. This makes identifying them crucial because some of these conditions require immediate intervention upon discovery. This could improve the patients’ quality of life.
